# Supplementary material for: Complexome profiling of the Chlamydomonas psb28 mutant reveals TEF5 as an early PSII assembly factor
Source: Plant Cell. 2025 Mar 17;37(6):koaf055. doi: 10.1093/plcell/koaf055 (PMC12164590; doi:10.1093/plcell/koaf055)
Supplement: koaf055_Supplementary_Data [file koaf055_supplementary_data.zip › psb28_interactive.zip!/plots/intensity/qualityPlots/peptides/ACS1_Cre01.g071662.t1.1.html]

### Cre01.g071662.t1.1

Trivial name: ACS1  
SeqCoverage [median]: 1.70  
Mass: 69.09 kDa  
  
 

| Sequence | MissedCleavage | Mass [Da] | wtIntensity | muIntensity | Score |
| --- | --- | --- | --- | --- | --- |
| VDDVLNVSGHR | 0 | 1209.61 | 31589.65 | 57860.73 | 119.76 |
|  |  |  | 31589.6 | 57860.7 |  |
